# Changes in L4/5 Intervertebral Foramen Bony Morphology with Age

**DOI:** 10.1038/s41598-018-26077-1

**Published:** 2018-05-16

**Authors:** Shuaifeng Yan, Kai Wang, Yunfan Zhang, Song Guo, Yan Zhang, Jun Tan

**Affiliations:** Department of Orthopedics, Shanghai East Hospital, Tongji University School of Medicine, Shanghai, 200120 China

## Abstract

The purpose of this study was to explore the morphological changes in L4/5 intervertebral foramen with age using a digital method. The closed boundaries of the intervertebral foramen (IGES) in different sagittal slices (inside, middle and outside) were obtained from Mimics, and then imported to a custom-written program, which provided quantitative distance between the nerve root and the closed curves. The quantitative information of each age group was used to produce radar chart and line chart for morphological and statistical analyses. Overall, the intervertebral foramen changes mainly occurred in the inner part from middle age to old age. The foraminal height decreased with age in the inside sagittal slice, while no significant difference was found in the middle sagittal slice or the outside sagittal slice. The foraminal width showed no decrease in each age group or each sagittal plane. The present study described foraminal geometry of asymptomatic males in different sagittal slices with age. This information enhances the knowledge of anatomical changes in intervertebral foramen with age, which provides better understanding of the pathology of intervertebral foramen diseases.

## Introduction

Low back pain (LBP) is a global health problem, causing enormous financial burden^1,2^. The lifetime prevalence of LBP is reported to be as high as 84%^3^. Radiculopathy is a common cause of low back and leg pain^4^. In general, radiculopathy caused by foramen stenosis consists of 8–11% nerve root compression^5^, and shows an increasing trend^6^. The bony intervertebral foramen is the doorway for the nerve root and its morphology is closely related to radiculopathy. Smaller intervertebral foramen and bony callus could cause radiculopathy^7,8^. Jeffrey M reported that the foraminal geometry was important in the pathology of spondylolisthesis, and its accurate and proper knowledge contributed to surgical treatment strategies in cases with foraminal stenosis^9^. Similar findings were observed in Matsumoto M *et al*.’s degenerative scoliosis study^10^. As a complex three-dimensional (3D) anatomical structure, any physiological or pathological risk factor that could change the morphology of intervertebral foramen may lead to radiculopathy. Whether the intervertebral foramen morphology changes with age remains unknown. The bony morphology of intervertebral foramen depends on the relative position of the neighboring anatomical structures, so previous studies^11–16^ on spine aging focused on intervertebral disc and facet joints due to their mobility. The relative position of adjacent vertebrae making up the bony boundary of intervertebral foramen significantly changes with the mobile structures including intervertebral disc and facet joints^11–14^, which leads to decrease in foraminal height and posterior vertebral body wedging^12,15–17^. However, there is limited knowledge about morphological changes of intervertebral foramen with aging. Though the boundary morphological characteristics of intervertebral foramen was described as oval, round and teardrop-shaped “window” in the lateral aspect of the lumbar spine^18^, stenotic changes of the foramen have also been observed^19–21^. The morphology of intervertebral foramen, whether and how it changes with aging, whether it simultaneously changes in each part of the intervertebral foramen with congruous tendency, and whether it changes gradually or suddenly with age remain unknown^11–16^. To the best of our knowledge, the relationship between aging and morphological change of intervertebral foramen has not been confirmed with a digital method. Hence, the purpose of this study was to explore this relationship.

The intervertebral foramen lies between the pedicles of neighboring vertebrae at all levels in the spine. The lumbar intervertebral foramen includes the adjacent vertebral pedicles superiorly and inferiorly, the postero-inferior margin of the superior vertebral body, the intervertebral disc, and the postero-superior vertebral notch of the inferior vertebral body anteriorly^22^. Many studies have focused on the morphology of intervertebral foramen *in vivo* and *in vitro*^20,23–26^. The foraminal height was reported as 11–23 mm and the foraminal width as 8–10 mm^4,18,23,25,27^. The foraminal height and width of intervertebral foramen were also examined in healthy subjects and patients with degenerative lumbar scoliosis, with the 3D method^10,24^. However, the quantitative statistical data was limited to the foraminal height, foraminal width and area of intervertebral foramen. Although there is no agreement on the morphology of intervertebral foramen, the variation may be caused by different methods adopted to determine the foraminal geometry^24^. Given that the intervertebral foramen is a complex 3D anatomical structure in vertical direction, it is impossible to directly describe its morphology. So in this study, a custom-written program was used to automatically and accurately obtain morphological and quantitative information of the intervertebral foramen. The purpose of the present study was to explore the morphological changes of the L4/5 intervertebral foramen with age.

## Patients and Methods

### Study participants

The sample comprised 25 asymptomatic male volunteers (Young Group: 28.8 ± 5.61 years; Middle Age Group: 47.67 ± 3.74 years; and Old Group: 69.17 ± 3.87 years), all of whom underwent lumbar spine CT (Volume Zoom, Siemens, Malvern, PA) at the Shanghai East Hospital. Exclusion criteria were: current or prior back pain, anatomical abnormalities, or any spinal disorders. The foramina of L4/5 was bilaterally measured in the participants. Shanghai East Hospital (East Hospital Affiliated to Tongji University) Medical Ethics Committee approved the study protocol, which met the relevant guidelines and regulations of Shanghai Medical Ethics Committee. All included volunteers had signed an informed consent form.

### Creation of the boundary closed curves of intervertebral foramen in different sagittal slices

**Figure 1:**
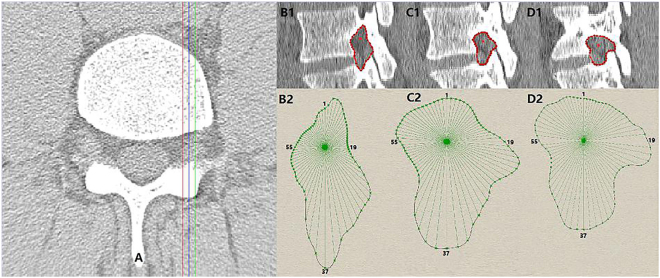
The process to reconstruct the boundary of intervertebral foramen. (**A**) Red line corresponds to the inside sagittal slice. Blue line corresponds to the middle sagittal slice. Green line corresponds to the outside sagittal slice.

The participants were scanned in the supine position. The CT images were imported into a reconstruction software (Mimics, Materialise, Inc., Leuven, Belgium). According to a study by Lee *et al*.^28^, the intervertebral foramen was subdivided into three zones: entrance zone, mid-zone and exit zone. Similarly, we chose three sagittal slices: the inside sagittal slice, the outside sagittal slice and the middle sagittal slice. In order to obtain the continuous boundary limit, a continuous line segment was used to represent the posterior margin of the intervertebral disc. Thus the boundary of intervertebral foramen is closed in any sagittal slice between the red line and the green line in Fig. 1A. We obtained the different closed curves of different boundaries of the intervertebral foramen in corresponding sagittal slices. Each boundary limit consisted of the superior and inferior pedicles, the postero-inferior margin of the superior vertebral body, the postero-superior margin of the inferior vertebral body, the superior articular facets and the inferior articular facets. The nerve root in the CT images is marked in Fig. 1. Figure 1(B1,C1 and D1) shows the boundary, and the red point in the closed curve is the center of the nerve root.

### Reconstructed boundary of intervertebral foramen

The closed curves of intervertebral foramen (IGES type file) in different sagittal planes obtained from Mimics were imported to a custom-written program (Rhino, Robert McNeel, Seattle, USA), and are shown in Fig. 1(B1,C2 and D2). Each curve was automatically divided into 72 equal parts, where the center of the nerve root serves as a pivot in the program. Thus, the angle between two line segments is 5°, and 1 and 37 represent the two line segments in vertical axis, while 19 and 55 in horizontal axis. The distance between the pivot and the boundary could be automatically determined for quantitative analysis.

### Radar charts and line charts of intervertebral foramen

**Figure 2:**
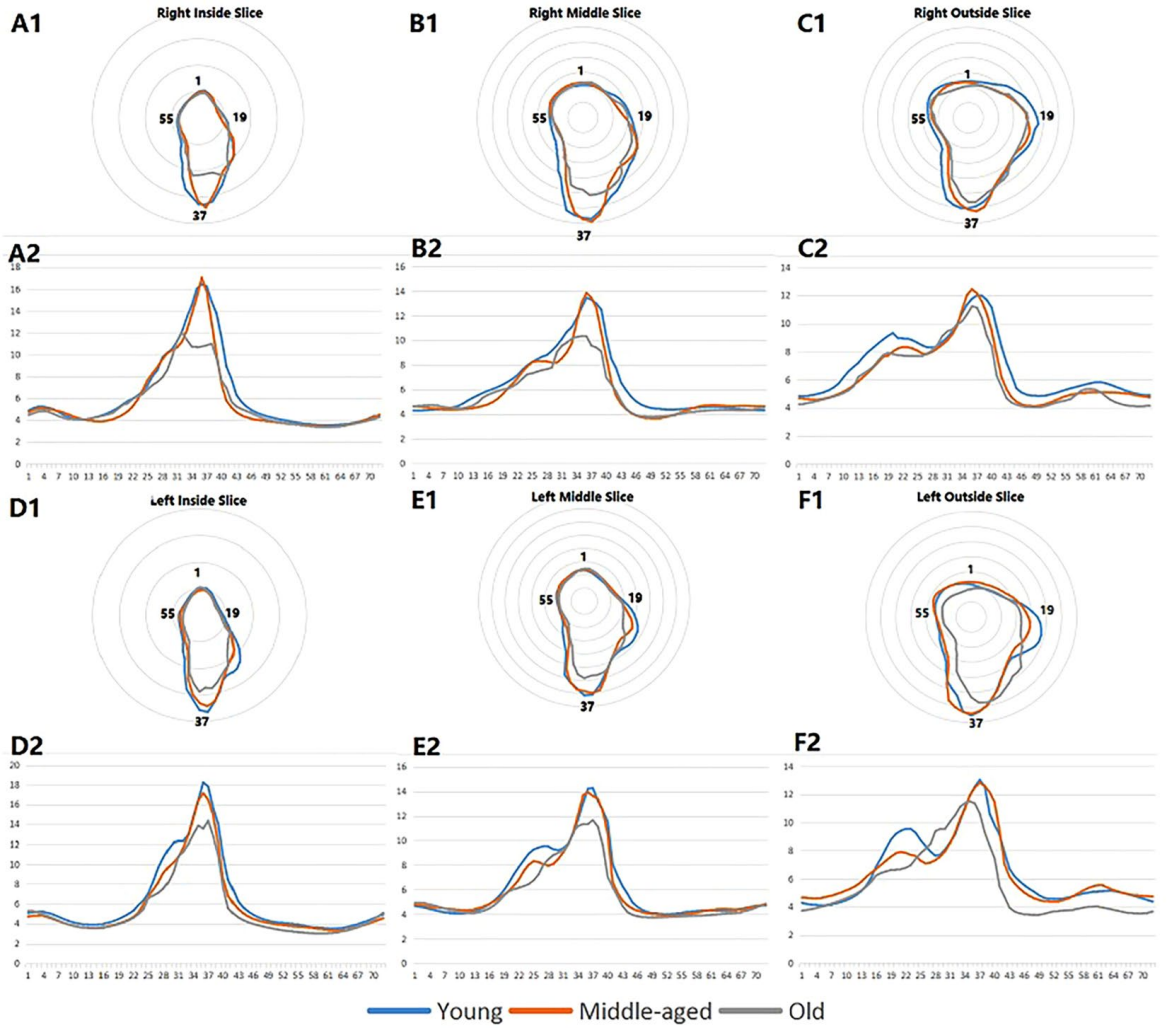
The radar charts of each sagittal plane for all age groups and the line charts of differential value of different age groups in each sagittal group. In the radar charts, Young: young group; Middle-aged: middle age group; Old: old age group. A1, B1, C1, D1, E1 and F1: The radar charts of each sagittal plane for all age groups. A2, B2, C2, D2, E2 and F2: The line charts of differential value of different age groups in each sagittal group. Right Inside Slice: the right inside sagittal slice of the L4/5 segment. Right Middle Slice: the right middle sagittal slice of the L4/5 segment. Right Outside Slice: the right outside sagittal slice of the L4/5 segment. Left Inside Slice: the left inside sagittal slice of the L4/5 segment. Left Middle Slice: the left middle sagittal slice of the L4/5 segment. Left Outside Slice: the left outside sagittal slice of the L4/5 segment. In the line charts, Young: the differential value between the young and middle age groups. Middle-aged: the differential value between the middle age and old age groups. Old: the differential value between the young and old age groups.

The data of different age groups exported from the custom-written program were averaged, and the averaged quantitative data were used to produce radar charts for morphological analysis. The closed curves from different age groups were compared in the same sagittal plane (Fig. 2A1,B1,C1,D1,E1 and F1). Figure 2(A2,B2,C2,D2,E2 and F2) shows the line charts of differential values for the three age groups in each sagittal group. In this study, we chose a range from 31 (150°) to 46 (225°) since the position of largest differential value of sagittal plane was located in this range. Foraminal height is defined as the vertical distance in orthogonal direction, while foraminal width is the horizontal distance. So the height is the distance from 1 to 37, and the width is the distance from 19 to 55.

### Statistical analysis

The distance between the different age groups in 31–46 (150°–225°) were compared for each sagittal plane by one-way ANOVA with F test. The foraminal height and width were compared in the different age groups for each sagittal plane by one-way ANOVA with F test. Significance level was set at p < 0.05. All data were reported as mean ± standard error of the mean (SEM).

## Results

### Morphological changes

Figure 2 shows the radar charts of each sagittal plane for all age groups. In the inside sagittal plane, the bilateral superior vertebral pedicle and the inferior articular process of the superior vertebra almost coincide with increase in age. As compared to the young and middle age groups, the distance from the nerve root to the inferior pedicle, and the intervertebral disc and superior articular process of the inferior vertebral body decreased, especially the distance between the nerve root and the inferior vertebral pedicle in the old age group. In the middle sagittal plane, the main decrease was seen in the lower part of the boundary including the distance from the nerve root to intervertebral disc, superior pedicle and the facet with aging. In the outside sagittal planes, the boundary of the old age group seemed smaller than the young and middle age groups from 1 to 72. Overall, the boundary of intervertebral foramen in different sagittal planes changed with aging. The result obtained from the left side differed from the right side.

### Distance between the nerve root and the boundary

In the line chart, we set the range from 31 (150°) to 46 (225°) as the quantitatively analyzed range because the position of largest differential value of each sagittal slice was located in the range (Fig. 2A2,B2,C2,D2,E2 and F2). Tables 1 and 2 show the statistical results in each angle of the range. The inside sagittal plane was significantly different in 37 on the left side and the 35–38 on the right side. There was no significant difference in any angle for the middle sagittal plane and the outside plane.

**Table 1:** Distance between the nerve root to the boundary in the range of 31–46 (Right side).

	Right Inside Sagittal Slice			Right Middle Sagittal Slice			Right Outside Sagittal Slice		
	Young (10)	Middle Age (9)	Old (6)	Young (10)	Middle Age (9)	Old (6)	Young (10)	Middle Age (9)	Old (6)
31	10.50 ± 2.45	10.39 ± 1.32	9.79 ± 2.29	9.59 ± 1.54	8.19 ± 1.42	9.04 ± 2.52	8.70 ± 1.09	8.49 ± 2.31	9.12 ± 2.71
32	11.21 ± 1.82	10.65 ± 1.31	11.26 ± 4.27	10.13 ± 1.69	8.54 ± 1.34	9.57 ± 3.19	9.01 ± 1.06	8.79 ± 2.22	9.44 ± 2.96
33	12.11 ± 1.42	11.17 ± 1.38	11.99 ± 4.92	10.70 ± 1.92	9.03 ± 1.20	9.82 ± 2.68	9.43 ± 1.06	9.22 ± 2.05	9.59 ± 2.85
34	13.42 ± 1.58	12.25 ± 1.73	11.34 ± 4.56	11.07 ± 1.71	9.74 ± 0.93	10.13 ± 2.65	9.97 ± 1.06	9.84 ± 1.80	9.92 ± 2.80
35	14.55 ± 1.65	13.75 ± 2.10	10.72 ± 4.03^*#^	11.80 ± 1.60	11.48 ± 1.31	10.28 ± 3.14	10.74 ± 1.29	10.99 ± 2.21	10.17 ± 2.96
36	16.04 ± 1.81	15.26 ± 2.27	10.67 ± 3.97^*#^	12.67 ± 1.90	13.07 ± 1.71	10.34 ± 3.42	11.35 ± 1.52	12.04 ± 2.69	10.65 ± 3.18
37	16.41 ± 2.42	17.04 ± 2.81	10.74 ± 4.62^*#^	13.46 ± 2.55	13.85 ± 2.52	10.32 ± 3.50	11.76 ± 1.61	12.43 ± 2.80	11.21 ± 3.80
38	16.23 ± 2.99	15.74 ± 4.65	10.85 ± 5.03^*#^	13.30 ± 3.10	13.48 ± 2.25	9.57 ± 4.55	11.99 ± 1.59	12.15 ± 2.95	11.13 ± 4.08
39	14.84 ± 4.61	12.85 ± 4.71	10.99 ± 6.58	12.99 ± 3.07	12.62 ± 1.99	9.42 ± 5.21	11.98 ± 1.53	11.57 ± 2.99	10.37 ± 4042
40	13.74 ± 4.75	9.96 ± 3.91	9.63 ± 6.19	12.50 ± 3.30	10.59 ± 2.92	9.10 ± 5.54	11.68 ± 1.49	10.70 ± 2.95	9.13 ± 5.15
41	11.51 ± 4.96	7.30 ± 2.45	7.62 ± 6.05	10.33 ± 4.09	8.63 ± 3.25	6.98 ± 4.72	11.14 ± 1.71	9.46 ± 2.88	8.38 ± 5.19
42	8.89 ± 4.29	5.76 ± 1.66	6.93 ± 5.53	8.53 ± 4.28	6.65 ± 2.27	6.43 ± 4.52	9.70 ± 2.56	7.75 ± 2.78	6.29 ± 3.48
43	7.78 ± 4.01	5.16 ± 1.33	5.68 ± 3.04	7.73 ± 4.10	5.48 ± 1.60	5.85 ± 3.85	8.34 ± 2.76	6.16 ± 1.44	5.48 ± 2.68
44	6.33 ± 1.76	4.73 ± 1.10	5.25 ± 2.31	6.56 ± 2.74	4.83 ± 1.09	5.05 ± 2.50	7.21 ± 2.90	5.32 ± 1.10	4.68 ± 1.46
45	5.70 ± 1.26	4.45 ± 1.00	5.02 ± 1.95	5.96 ± 2.23	4.40 ± 0.83	4.46 ± 1.59	6.34 ± 2.75	4.76 ± 0.88	4.38 ± 1.17
46	5.26 ± 0.97	4.26 ± 0.97	4.84 ± 1.67	5.44 ± 1.76	4.10 ± 0.70	4.13 ± 1.24	5.38 ± 1.52	4.44 ± 0.80	4.22 ± 1.04

*p < 0.05, compared to the young group.

^#^p < 0.05, compared to the middle age group.

**Table 2:** Distance between the nerve root to the boundary in the range of 31–46 (Left side).

	Left Inside Sagittal Slice			Left Middle Sagittal Slice			Left Outside Sagittal Slice		
	Young (10)	Middle Age (9)	Old (6)	Young (10)	Middle Age (9)	Old (6)	Young (10)	Middle Age (9)	Old (6)
31	12.18 ± 2.34	10.20 ± 2.40	9.20 ± 2.50	9.21 ± 1052	8.51 ± 1.04	8.90 ± 2.51	8.10 ± 1.71	7.98 ± 1.55	9.58 ± 1.69
32	12.36 ± 2.14	10.77 ± 1.88	10.84 ± 4.05	9.37 ± 1.57	9.05 ± 1.17	9.20 ± 2.31	8.62 ± 1.92	8.55 ± 1.67	10.09 ± 2.33
33	12.38 ± 1.95	11.74 ± 1.19	11.29 ± 3.96	9.75 ± 1.70	9.69 ± 1.35	9.69 ± 2.28	9.18 ± 2.08	9.31 ± 1.76	10.54 ± 2.77
34	12.97 ± 2.35	12.90 ± 1.67	12.05 ± 4.38	10.59 ± 1.97	10.69 ± 1.62	10.72 ± 2.15	10.12 ± 2.42	10.27 ± 1.65	11.13 ± 2.82
35	14.83 ± 3.54	14.62 ± 2.58	13.00 ± 3.72	11.73 ± 2.50	12.14 ± 1.89	11.19 ± 2.43	11.10 ± 2.69	11.00 ± 1.34	11.39 ± 2.87
36	16.49 ± 3.48	16.43 ± 2.33	13.87 ± 2.0	13.03 ± 2.61	13.69 ± 1.23	11.33 ± 2.65	11.93 ± 2.62	11.97 ± 1.34	11.51 ± 2.93
37	18.26 ± 3.65	17.16 ± 2.37	13.57 ± 2.29^*#^	14.21 ± 2.58	13.92 ± 1.46	11.35 ± 2.65	12.58 ± 2.04	12.51 ± 1.44	11.36 ± 3.50
38	17.85 ± 3.31	16.51 ± 3.35	14.36 ± 5.28	14.28 ± 2.60	13.64 ± 1.74	11.66 ± 3.32	13.04 ± 1.79	12.83 ± 1.87	10.64 ± 4.19
39	15.70 ± 4.40	14.94 ± 5.39	12.84 ± 6.26	13.22 ± 3.49	13.39 ± 2.97	11.14 ± 4.18	12.56 ± 1.96	12.62 ± 2.45	9.30 ± 5.19
40	14.09 ± 3.88	12.17 ± 6.17	10.94 ± 6.05	12.45 ± 4.16	12.38 ± 3.92	9.60 ± 5.52	10.61 ± 3.78	12.16 ± 3.24	8.34 ± 5.75
41	10.86 ± 3.85	8.58 ± 2.97	7.65 ± 3.68	11.53 ± 3.92	10.53 ± 4.37	7.05 ± 4.94	9.82 ± 4.20	11.45 ± 4.35	7.44 ± 5.81
42	8.44 ± 2.57	6.81 ± 1.76	5.55 ± 0.95	8.10 ± 3.09	6.62 ± 1.47	6.21 ± 4.70	9.13 ± 4.23	9.42 ± 2.76	5.46 ± 3.86
43	7.39 ± 2.31	6.05 ± 176	4.98 ± 0.76	7.12 ± 2.80	5.75 ± 1.18	5.45 ± 4.03	7.89 ± 3.62	7.05 ± 1.77	4.72 ± 2.98
44	6.21 ± 1.01	5.45 ± 1.27	4.60 ± 0.68	6.30 ± 2.47	5.12 ± 1.02	4.68 ± 2.67	6.73 ± 2.87	6.16 ± 1.52	3.95 ± 1.63
45	5.62 ± 0.77	5.02 ± 1.13	4.32 ± 0.66	5.68 ± 2.09	4.73 ± 0.96	4.18 ± 1.75	6.20 ± 2.56	5.69 ± 1.34	3.68 ± 1.27
46	5.20 ± 0.66	4.70 ± 0.99	4.10 ± 0.66	4.98 ± 1.46	4.48 ± 0.93	3.96 ± 1.40	5.85 ± 2.34	5.32 ± 1.18	3.55 ± 1.11

^*^p < 0.05, compared to the young group.

^#^p < 0.05, compared to the middle age group.

### Foraminal height and width

Overall, foraminal height decreased in each sagittal plane with age (the young group and the old age group, p < 0.05; the middle age group and the old age group, p < 0.05; the young group and the middle age group, p > 0.05), while the foraminal width did not show significant difference in each age group. Table 3 shows the quantitative results of foraminal height and width.

**Table 3:** Bilateral foraminal height and width in each sagittal slice for all age groups.

	Height			Width		
	Young (10)	Middle Age (9)	Old (6)	Young (10)	Middle Age (9)	Old (6)
Right Inside Sagittal Slice	21.31 ± 3.11	21.76 ± 3.70	15.20 ± 5.71^*#^	8.75 ± 3.29	7.71 ± 1.34	8.30 ± 1.46
Left Inside Sagittal Slice	23.31 ± 3.69	21.85 ± 2.98	18.69 ± 3.12^*#^	8.16 ± 2.26	7.55 ± 1.38	6.94 ± 1.32
Right Middle Sagittal Slice	17.76 ± 2.47	18.49 ± 3.38	14.95 ± 4.10	10.59 ± 3.63	9.23 ± 1.61	9.85 ± 1.70
Left Middle Sagittal Slice	18.89 ± 2.26	18.70 ± 1.90	16.30 ± 3.52	9.58 ± 1.52	9.24 ± 1.26	8.92 ± 1.28
Right Outside Sagittal Slice	16.60 ± 1.37	17.11 ± 2.71	15.47 ± 4.02	14.04 ± 3.72	12.32 ± 2.27	12.21 ± 2.00
Left Outside Sagittal Slice	16.87 ± 2.22	17.14 ± 1.73	15.10 ± 4.16	12.78 ± 2.65	11.79 ± 1.76	10.29 ± 1.85

*p < 0.05, compared to the young group.

^#^p < 0.05, compared to the middle age group.

## Discussion

**Figure 3:**
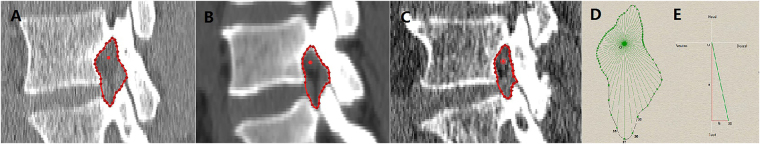
The intervertebral foramen morphology of different age groups in the right inside slice and exploded view of “35”. (**A**) morphology of intervertebral foramen in a young subject. (**B**) morphology of intervertebral foramen in a middle-aged subject. (**C**) morphology of intervertebral foramen in an old subject. (**D**) IGES of the divided boundary in the right sagittal inside slice. The distance of “35/36/37/38” is significantly different in different age groups. O is the center of nerve root. (**E**) 35 in the right inside slice was divided into a in the head-foot direction and b in the ventro-dorsal direction. O is the center of nerve root.

The changes in intervertebral foramen morphology mainly occur in the inter part and boundary in the ventro-dorsal direction, which has an adduction tendency in all sagittal slices. The bony morphology of the intervertebral foramen showed no change from young to middle age, but obviously decreased in the old age group as compared to the young and middle age groups in each sagittal slice. The bony morphology of the intervertebral foramen appears to change with age. The morphological changes of inner part boundary of intervertebral foramen were obvious in different age groups (Fig. 3A–C). The distance between the nerve root and the boundary was first described every 5°, based on the nerve root as the center. In the inside sagittal slice, the statistical difference was not the same on both sides: 35–38 in the right and 37 in the left, which may be due to the degree of degeneration. Using 35 as an example, in Fig. 3E, we divided 35 from the right inside sagittal slice as a in the head-foot direction and b in the ventro-dorsal direction. The statistical difference of 35 could be viewed as difference between a and b. The decrease of a in the head-foot direction may be caused by many risk factors such as the decrease of the intervertebral disc, the fracture of the vertebral body, etc. The statistical difference of b represents the decrease of distance in the ventro-dorsal direction, which could be affected by osteophyte. As compared to the young and middle aged groups (Fig. 3A and B), the adduction tendency and the effect of decrease in intervertebral disc was seen in the morphology of intervertebral foramen in the old age group (Fig. 3C). In the middle and outside sagittal slices, no statistical difference existed in any angle of the boundary. The decrease of foraminal height was observed in the inside sagittal slice with age, while the foraminal width seemed stable with age. However, Senoo *et al*. showed age-related foraminal height and width decrease in healthy subjects^24^. Such differences may be attributed to the validation of the adopted method.

Intervertebral foramen is a composite structure consisting of bone and ligamentum flavum. The soft tissue structures such as intervertebral disc and ligaments were considered to be important in the pathology of diseases related to intervertebral foramen^29,30^. However, since the reconstructed intervertebral foramen was limited to the bony threshold value, we analyzed the morphology of the foramen without the soft tissue in the present study. In order to obtain more knowledge on the intervertebral foramen, soft tissue should be taken into consideration in future studies. The other limitation is that the primary data of the CT scan was collected only in the supine position, without dynamic and axial positions. The morphology of intervertebral foramen would change with position. Iwata *et al*.^26^ demonstrated the effects of axial loading on the lumbar foraminal geometry *in vivo*. Zhong *et al*.^31^ demonstrated the dynamic changes of dimensions in the lumbar intervertebral foramen. Although the results presented in this study showed significant morphological differences with age in the supine position, the morphological changes in physiological loading position and dynamic position should also be examined for better understanding of the pathology of intervertebral foramen diseases.

In summary, the present study described how foraminal geometry changes with age. Although the geometry is limited to the bony structures in males, this information may be valuable for better understanding intervertebral foramen diseases.

### Ethical approval

Shanghai East Hospital (East Hospital Affiliated to Tongji University) Medical Ethics Committee approved the study protocol, which met the relevant guidelines and regulations of Shanghai Medical Ethics Committee. All included volunteers had signed an informed consent form.

### Data availability

The datasets analyzed in the current study are available from the corresponding author upon request.
